# Deprescribing—A Few Steps Further

**DOI:** 10.3390/pharmacy6040112

**Published:** 2018-10-11

**Authors:** Cheryl A. Sadowski

**Affiliations:** Faculty of Pharmacy & Pharmaceutical Sciences, University of Alberta, Edmonton, AB T6G 1C9, Canada; cherylas@ualberta.ca

While polypharmacy had been discussed for decades in the geriatrics literature, it has taken on new urgency as we deal with population aging, increased medication use, and frequent adverse drug events [1]. One obvious solution, deprescribing, has only been studied for approximately ten years, with the term first appearing in the literature in 2003 [2]. This special issue in *Pharmacy* highlights the growing international research on deprescribing. Not only is it relevant to geriatrics, but patient populations with multimorbidity, often sentenced to live with long and complex medication regimens, are being studied.

The ideal setting for deprescribing is something that has not been confirmed, although many experts in this field would argue that any time an unnecessary or potentially inappropriate medication can be removed, it should be done. In this issue, there are papers showing that health professionals believe that deprescribing can take place in acute care settings, nursing homes can implement a benzodiazepine deprescribing protocol, a hemodialysis unit can be supported by pharmacists to identify medications for deprescribing, and deprescribing in the community maintains patients’ trust [3,4,5,6]. Special populations are also considered in this special issue, including those undergoing hemodialysis, those with cancer, and adults with intellectual disabilities [3,7,8].

Most research on deprescribing has focused on potentially inappropriate and high-risk medications, such as benzodiazepines. In this issue Javelot and colleagues expand this research into nursing homes, while studying the outcomes of the deprescribing. In landmark studies, we have evidence that deprescribing can be done, such as in the EMPOWER study where a patient education intervention led to a 27% reduction in benzodiazepines at 6 months in the intervention group [9]. However, this and other studies did not include the outcomes that would demonstrate negative patient outcomes (e.g., poorer sleep quality, increased anxiety), clinical outcomes (e.g., falls), or health system outcomes (e.g., emergency department visits, hospitalizations). It is assumed that deprescribing is positive, particularly for benzodiazepines, because these medications are associated with falls, cognitive impairment, and sedation, and because their harm outweighs their benefit in most seniors, are listed on the Beers Criteria [10]. But without studies such as Javelot, we cannot be so confident. Javelot presents the initial analysis of a nursing home study where, despite a small sample size, those who had their benzodiazepines withdrawn, compared to those who remained on their benzodiazepines, had no difference in falls at the end of the tapering phase, but had fewer falls at the 1-year follow-up [5]. We also learn from Javelot that a tapering protocol for benzodiazepines over 4–10 weeks was implemented. This sample of patients was a mean of 89 years of age, indicating that a patient population is never too old to address high risk medication use. At 1 year, 36% of patients had persisted without their benzodiazepines [5].

Deprescribing is not just a concern for geriatrics. The study on hemodialysis patients included a sample that was, on average, 63 years old, had 3 comorbidities, and were taking 14 different medications [3]. In this 3 month study of pharmacist medication review, 36% of patients had a medication without an indication, and overall the pharmacist made 3 recommendations/patient for a medication change, with 1 being implemented. One barrier found to implementing the pharmacist recommendations in this study was the referral to other specialists outside of nephrology to reconsider the therapeutic management of particular conditions [3]. This study helps us appreciate that the dynamic of specialists versus primary care, or physicians versus pharmacists, remain challenges to decision making regarding medications. In this study specialists working with other specialists can create a dynamic where there is lack of clarity on how to address identified medication overuse [3].

A study from the UK also highlights the importance of determining roles and expectations for health professionals, to ensure that medication reviews and deprescribing are handled in a systematic way, and to avoid professional conflicts to the detriment of patient care [4]. This innovative team used a consensus approach with 10 scenarios to establish generalised guidance for the pharmacist to lead the medication review and deprescribing [4]. They built on the familiar tools of STOPP and regional polypharmacy guidance documents [11]. They considered the documentation of frailty, use of the electronic health records, and multimorbidity and various age cut-offs. This type of research is challenging, time-consuming, but extremely valuable for grey, subtle, nuanced areas of clinical practice. They found the experts emphasized that pharmacists and physicians both play a role, and face to face communication was particularly valuable. Identifying the delicate nature of stopping another health professional’s order, the team suggested that in some urgent cases the pharmacist could ‘hold’ the medication, but not ‘deprescribe’ it, an activity that should only be done by the ‘prescriber’. This still leaves the question unanswered regarding a non-physician prescriber (e.g., pharmacist, nurse practitioner), and guidance regarding the relationship and respect about who can stop those prescriptions. The focus of this research was on protocol and clear roles, in order to optimize patient care, which is appropriate in a hierarchical setting such as a British hospital, but the principles can apply elsewhere. Negotiating good patient care requires collaboration, respect, boundaries, and fulfilling expectations, to effectively work as a team, and health professionals who exercise a full scope of practice, without awareness or regard for the nuances of collaboration, will do so to the detriment of patient care.

Goncalves also highlights the challenges of deprescribing in cancer patients, noting that even in this population with a predictable clinical trajectory, physicians may be hesitant to deprescribe if a specialist ordered the medication [7]. Other barriers to deprescribing have been noted in older adults with limited life expectancy, and a simplistic algorithm is not appropriate, given that life expectancy in some cancers may be measured in years, and preventive and disease managing drugs should continue to be used to optimize health [12,13]. This review builds on Goncalves previous work in finding that the many chronic medications were deemed futile in patients referred to palliative care [14]. He also notes the importance of dialogue and collaboration with nurses and pharmacists, while highlighting tools and criteria that can be used to guide clinicians in having discussions with patients regarding deprescribing, and even proposes a 6-step process for deprescribing in cancer patients, that can be validated in future studies [7].

The focus on special populations is particularly important. A commentary on those with intellectual disabilities is timely, given the focus on the use of antipsychotics for challenging behaviours in this population [8]. Individuals with intellectual disabilities often have a high prevalence of chronic conditions, such as heart disease and diabetes, yet have difficulty accessing health care and resources [15]. Flood calls to action the pharmacists, front-line care workers, and physicians, who all contribute to overuse of psychotropic medications, and cautions against the use of medication appropriateness tools or criteria, as there has been little effort to validate these in patients with intellectual disability. This population of adults is particularly vulnerable, as they often cannot consent to treatment, and communication challenges can delay identification of adverse drug events, yet research often excludes these patients and the evidence base for treatment is not robust for intellectual disabilities [8,15]. This commentary highlights unique barriers to deprescribing in this population, including the lack of training for health care professionals, transitions between community and institutional settings for these patients, lack of documentation for deprescribing attempts, transition from childhood to adulthood, biases of the public and health professionals, and limited access to specialists psychiatrists, to name a few [8].

The studies in this issue also highlight the importance of studying process. Both Flood and Goncalves provide steps to consider in medication assessment and deprescribing, Marvin et al. suggest a structured approach with transparency for healthcare professionals, and Alshamrani describes a framework for pharmacists to use in identifying some of the medication-related problems [3,4,7,8]. In one final paper, Zhang et al. assess the impact of a patient-focused intervention on the relationship and trust those patients have with their healthcare providers [6]. There have been concerns that patient barriers to deprescribing can include relationship and communication issues with health professionals [16]. This paper delves into this topic, recognizing that patient education, without healthcare professional leadership, can still lead to older adults advocating and reducing their use of benzodiazepines [9]. However, this approach challenges the power dynamic usually held in a physician-patient relationship. This study involved the Primary Care Assessment Survey questions about trust, with patients responding on a 5-point Likert scale (strongly agree to strongly disagree). The overall trust at baseline showed higher level of complete trust in physicians versus pharmacists, but throughout the 6 months of intervention, there was no change in level of trust for either physicians or pharmacists, demonstrating that patient education is an important method of intervention, that does not compromise the relationship patients already have established with their physicians and pharmacists.

While the challenges with deprescribing often seem daunting—changing the healthcare system, pharmacy reimbursement, professional practices, patient expectations—some of the studies included in this issue show that we are moving forward, and that small steps in our practice settings can be taken to increase the integration of deprescribing. There are still many important questions yet to be answered. Will there be tools that can guide us through the grey areas of risk versus benefit of chronic medications? What are the roles and boundaries for health professionals? How do we address overuse of medication and deprescribing in special and vulnerable populations? Fortunately, the research is continuing to guide us further on this journey to optimize the safe use of medications for older adults, vulnerable populations, and the increasing population of multimorbid patients.
